# Improved Accuracy for the Sturm-Pastyr Localizer in the Presence of Image Noise

**DOI:** 10.7759/cureus.17905

**Published:** 2021-09-12

**Authors:** Armando L Alaminos-Bouza, Russell A Brown

**Affiliations:** 1 Medical Physics, MEVIS Informática Médica Ltda., São Paulo, BRA; 2 Principal Engineer, Retired, Palo Alto, USA

**Keywords:** image-guided stereotactic surgery, stereotactic frame, frame-based stereotactic surgery, image-guided radiosurgery, monte-carlo simulation, sturm-pastyr localizer, mri- magnetic resonance imaging, computed tomography (ct ), stereotactic radiosurgery srs, stereotactic surgery

## Abstract

Image guidance for frame-based stereotaxis is facilitated by incorporating three to four Sturm-Pastyr (SP) localizers into a stereotactic frame. Typically, each SP localizer enables the calculation of one set of $\left ( x,y,z \right )$ coordinates in the three-dimensional coordinate system of the stereotactic frame, given three sets of $\left ( u, v \right )$ coordinates created by the SP localizer in the two-dimensional coordinate system of a computed tomography (CT) image. Bouza and Brown propose formulas to calculate three sets of $\left ( x,y,z \right )$ coordinates for each SP localizer. Monte Carlo (MC) simulation compares the accuracy of the new formulation to the accuracy of the original SP formulation that calculates only one set of $\left ( x,y,z \right )$ coordinates for each SP localizer. Monte Carlo simulation reveals that the calculation of three sets of $\left ( x,y,z \right )$ coordinates instead of only one set improves the accuracy of image guidance.

## Introduction

Image guidance for frame-based stereotaxis is facilitated by incorporating three to four Sturm-Pastyr (SP) localizers [1-3] or N-localizers [4] into a stereotactic frame. Typically, each SP localizer enables the calculation of one set of $\left ( x,y,z \right )$ coordinates in the three-dimensional (3D) coordinate system of the stereotactic frame, given three sets of $\left ( u, v \right )$ coordinates created by the SP localizer in the two-dimensional (2D) coordinate system of a computed tomography (CT) image [2,3]. Hence, for three or four SP localizers, three or four sets of $\left ( x,y,z \right )$ coordinates are calculated respectively. Monte Carlo simulation predicts that for other types of localizers, more than three sets of $\left ( x,y,z \right )$ coordinates improve the accuracy of image guidance [5,6]. This article reports the calculation of three sets of $\left ( x,y,z \right )$ coordinates for each SP localizer instead of only one set; hence, for three or four SP localizers, nine or 12 sets of $\left ( x,y,z \right )$ coordinates are calculated respectively. These nine or 12 sets of $\left ( x,y,z \right )$ coordinates improve the accuracy of image guidance without requiring any modification to the SP localizer.

## Technical report

**Figure 1:**
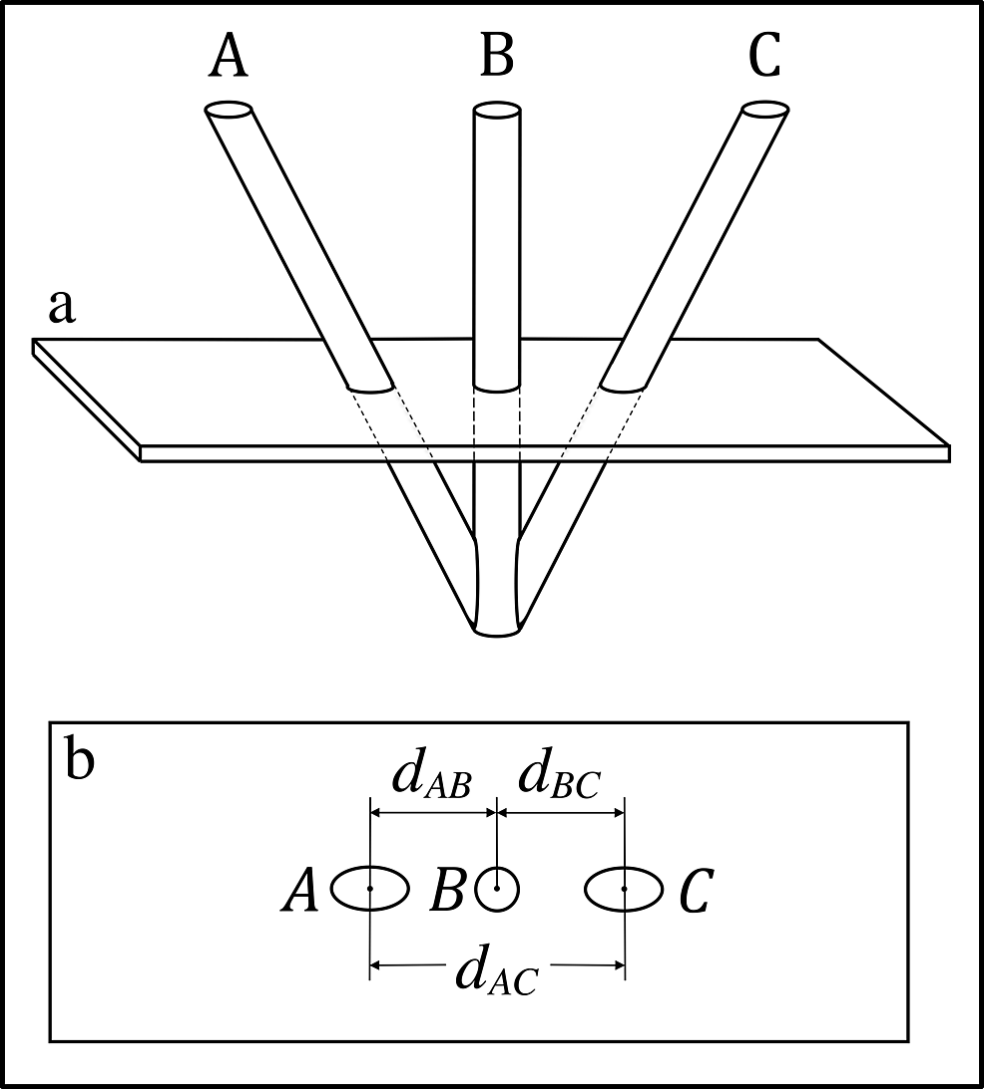
The Sturm-Pastyr localizer and its intersection with a CT scan slice $\left( \mathrm a \right)$ Side view of the Sturm-Pastyr localizer. A CT scan slice intersects rods $\mathrm A$, $\mathrm B$, and $\mathrm C$. $\left( \mathrm b \right)$ CT scan image. The intersection of the CT scan slice with rods $\mathrm A$, $\mathrm C$, and $\mathrm B$ creates fiducial ellipses $A$ and $C$ and fiducial circle $B$ respectively in the CT scan image [3]. The distances $d_{AB}$, $d_{BC}$, and $d_{AC}$ between the $\left( u,v \right)$ coordinates of the centers of fiducials $A$, $B$, and $C$ in the 2D coordinate system of the CT scan image enable the calculation of the $\left( x,y,z \right)$ coordinates of the points of intersection of the CT scan plane with the cylindrical axes of rods $\mathrm A$, $\mathrm B$, and $\mathrm C$ in the 3D coordinate system of the stereotactic frame [2,3]. The appendices provide details of this calculation.

Figure 1 depicts the Sturm-Pastyr (SP) localizer that comprises two diagonal rods $\mathrm A$ and $\mathrm C$ and one vertical rod $\mathrm B$. The cylindrical axes of these three rods are coplanar. The intersection of a CT scan slice with rods $\mathrm A$, $\mathrm C$, and $\mathrm B$ creates fiducial ellipses $A$ and $C$ and fiducial circle $B$ respectively in the CT scan image [3]. These three fiducials facilitate the transformation of the $\left( u,v \right)$ coordinates of a target point defined in the 2D coordinate system of the CT image into $\left( x,y,z \right)$ coordinates in the 3D coordinate system of the stereotactic frame [2,3]. In the absence of image noise, the $\left( u,v \right)$ coordinates of the centers of the fiducials are colinear. Hence, the Euclidean distances (*aka* Pythagorean distances) $d_{AB}$, $d_{BC}$, and $d_{AC}$ between the centers of the fiducials are linearly dependent and are related by the equation $d_{AC} = d_{AB} + d_{BC}$. However, image noise randomly perturbs the $\left( u,v \right)$ coordinates of the centers of the fiducials such that these centers are not colinear. In that case, the distances $d_{AB}$, $d_{BC}$, and $d_{AC}$ are linearly independent and $d_{AC} \neq d_{AB} + d_{BC}$. The linear independence of these three distances is exploited by the mathematics presented in the appendices.

**Figure 2:**
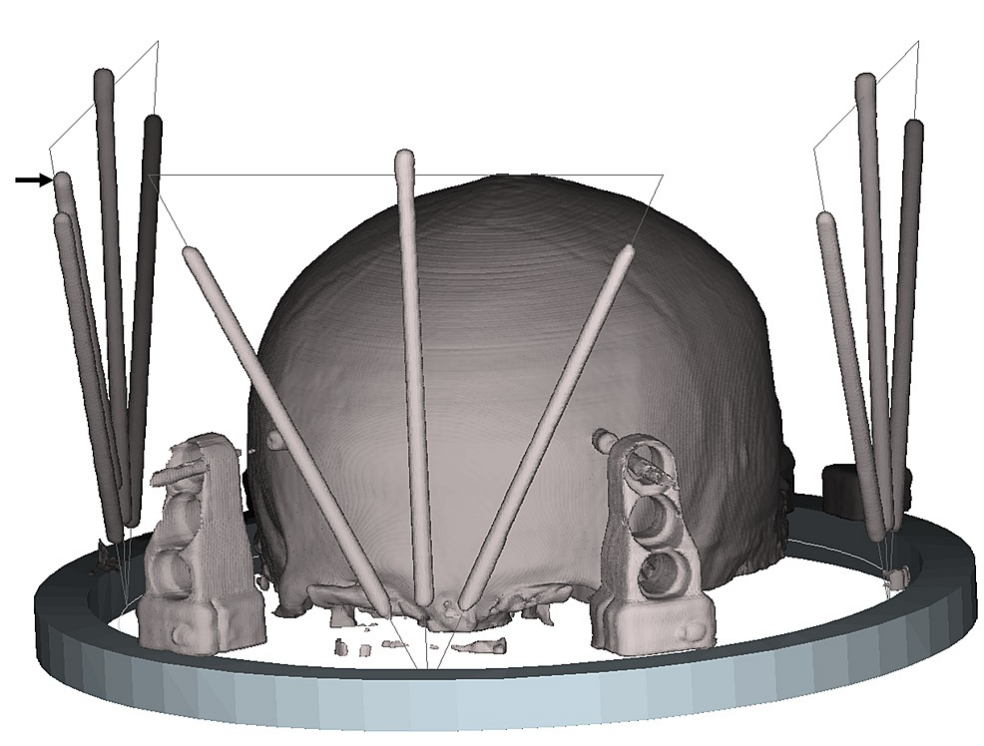
Three Sturm-Pastyr localizers attached to a Zamorano-Dujovny stereotactic frame A 3D reconstruction from a series of CT scan images shows three Sturm-Pastyr (SP) localizers attached to a Zamorano-Dujovny (ZD) stereotactic frame. The SP localizer attached to the right lateral aspect of the ZD frame includes an additional diagonal rod designated by the arrow. This rod produces in each CT scan image an ellipse that is not used to calculate $\left( x,y,z \right)$ coordinates but instead identifies the right lateral SP localizer in that CT scan image.

Figure 2 depicts the attachment of one SP localizer to each of the anterior, left lateral, and right lateral aspects of a Zamorano-Dujovny (ZD) stereotactic frame (Inomed Medizintechnik GmbH, Emmendingen, Germany). The three SP localizers create nine fiducials in the CT scan image. These nine fiducials are sufficient to determine the 3D spatial orientation of the CT scan plane relative to the ZD frame. Typically, only one set of $\left( x,y,z \right)$ coordinates is calculated from the fiducials created by each of the three SP localizers, for a total of three sets of $\left( x,y,z \right)$ coordinates [2,3]. However, the 3D spatial orientation of the CT scan plane may be determined to greater accuracy if three sets of $\left( x,y,z \right)$ coordinates are calculated for each SP localizer via equations (10-12) presented in the appendices. The resulting nine sets of $\left( x,y,z \right)$ coordinates may then be used to create an overdetermined system of linear equations that are solved via minimization of the least-square error to obtain a three by three transformation matrix that specifies the 3D spatial orientation of the CT scan plane relative to the ZD frame [2,5].

Accuracy is evaluated via Monte Carlo (MC) simulation that calculates a root mean square error (RMSe). The details of MC simulation have been described previously [6] and are summarized as follows. MC simulation performs $2^{21}\approx 2$ million iterations at each of numerous heights $\left( z \right)$, where $z$ is incremented by 2mm throughout the vertical extent of the SP localizer. At each height, random noise that has a maximum magnitude of 1.0mm perturbs the $\left( u,v \right)$ coordinates of the centers of the fiducials. The unperturbed and randomly perturbed centers of the fiducials are used to construct unperturbed and perturbed three by three matrices respectively. These unperturbed and perturbed matrices transform the $\left(u,v\right)$ coordinates of five target points from the 2D coordinate system of the CT scan image into the 3D coordinate system of the ZD frame to obtain unperturbed and perturbed $\left(x,y,z\right)$ coordinates respectively for each target point. The target points, whose pre-transformed $\left(u,v\right)$ coordinates are expressed in mm relative to the center of the CT scan image, are located at center $\left(0,0\right)$; right lateral $\left(+50, 0\right)$; left lateral $\left(-50,0\right)$; anterior $\left(0,+50\right)$; posterior $\left(0,-50\right)$; and anterolateral $\left(+50,+50\right)$. For each iteration and each target point at each height $\left( z \right)$, the squared 3D Euclidean distance between the unperturbed and perturbed target point is summed. After two million iterations, the RMSe is calculated from the sum for each target point [6] 

$$\mathrm{RMSe} = \sqrt{ \frac{1}{n} \sum_{i}^{n} \left[ \left ( x_i - \hat x_i \right )^2 + \left ( y_i - \hat y_i \right )^2 + \left ( z_i - \hat z_i \right )^2 \right] } \;\;\;\;\;\; \left ( 1 \right )$$

where $\left(x_i,y_i,z_i\right)$ and $\left( \hat x_i, \hat y_i, \hat z_i\right)$ are the respective unperturbed and perturbed $\left(x,y,z\right)$ coordinates for each iteration $i$ and where $n = 2^{21}$.

The results of the MC simulation are presented as follows.

## Discussion

Maximum accuracy for the Sturm-Pastyr (SP) localizer is achieved when the CT scan plane is perpendicular to the vertical rod $\mathrm B$ of the SP localizer, i.e., when the base of the stereotactic frame is parallel to the CT scan plane. Any tilt of the base of the stereotactic frame that disrupts this parallel orientation degrades accuracy [3]. For this reason, MC simulation to predict the RMSe has been performed for CT scan planes tilted relative to the base of the Zamorano-Dujovny (ZD) stereotactic frame at angles of $0^{\large\circ}$ and $10^{\large\circ}$ in the anteroposterior dimension. For tilt in this dimension, the height $\left( z \right)$ of the CT scan plane relative to the base of the ZD frame is greater anteriorly than posteriorly.

**Figure 3:**
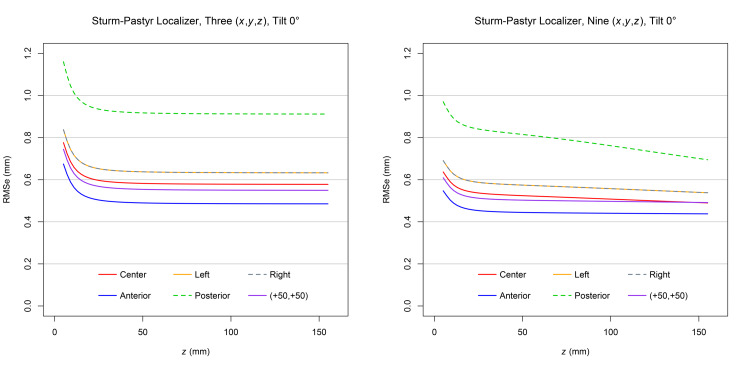
Plots of RMSe vs height $\left( z \right)$ at a $0^{\large \circ}$ tilt for three and nine sets of Sturm-Pastyr $\left( x,y,z \right)$ coordinates The RMSe for three sets of $\left( x,y,z \right)$ coordinates exceeds the RMSe for nine sets of $\left( x,y,z \right)$ coordinates at each target point for the range of heights $5.0 \mathrm{mm} \le z \le 155.0 \mathrm{mm}$. The plots for the left and right target points are superimposed. RMSe: Root mean square error

Figure 3 demonstrates that for each target point and at a $0^{\large\circ}$ tilt, the RMSe predicted for three sets of $\left( x,y,z \right)$ coordinates exceeds the RMSe predicted for nine sets of $\left( x,y,z \right)$ coordinates. The RMSe for the posterior target point exceeds the RMSe for all other target points because an SP localizer is attached to each of the anterior, left lateral, and right lateral aspects of the ZD frame but not to its posterior aspect [7]. The RMSe for the left and right target points are equal, as expected for SP localizers positioned symmetrically at the left and right aspects of the ZD stereotactic frame. For each target point, the RMSe increases significantly as the height $\left( z \right)$ approaches 0.0mm inferiorly near the apices of the V-shaped SP localizers [3].

**Figure 4:**
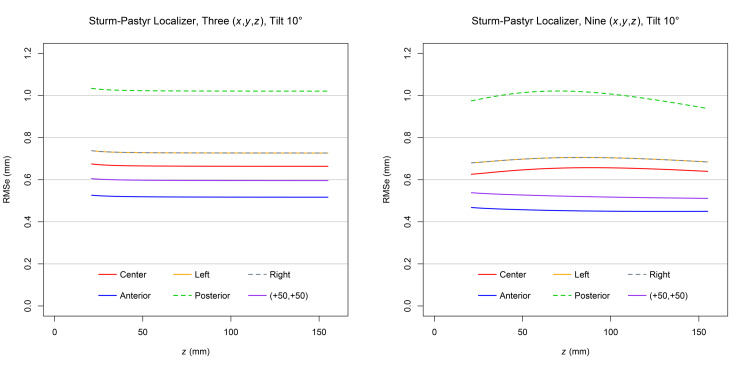
Plots of RMSe vs height $\left( z \right)$ at a $10^{\large \circ}$ tilt for three and nine sets of Sturm-Pastyr $\left( x,y,z \right)$ coordinates The RMSe for three sets of $\left( x,y,z \right)$ coordinates exceeds the RMSe for nine sets of $\left( x,y,z \right)$ coordinates at each target point for the range of heights $21.0 \mathrm{mm} \le z \le 155.0 \mathrm{mm}$. The plots for the left and right target points are superimposed. RMSe: Root mean square error

Figure 4 demonstrates that for each target point and at a $10^{\large\circ}$ tilt, the RMSe predicted for three sets of $\left( x,y,z \right)$ coordinates exceeds the RMSe predicted for nine sets of $\left( x,y,z \right)$ coordinates. Comparison to Figure 3 reveals that for each target point, the RMSe for a $10^{\large\circ}$ tilt exceeds the RMSe for a $0^{\large\circ}$ tilt. The RMSe for the posterior target point exceeds the RMSe for all other target points. The minimum allowed height $\left( z \right)$ of the CT scan plane is greater for a $10^{\large\circ}$ tilt than for a $0^{\large\circ}$ tilt so that the CT scan plane does not intersect the base of the ZD stereotactic frame posteriorly.

In view of the large RMSe for the posterior target point, MC simulation has been performed for the optional configuration of the ZD stereotactic frame that includes a fourth SP localizer attached to the posterior aspect of the frame. This configuration allows calculation of either four or 12 sets of $\left( x,y,z \right)$ coordinates via equations (10-12) presented in the appendices.

**Figure 5:**
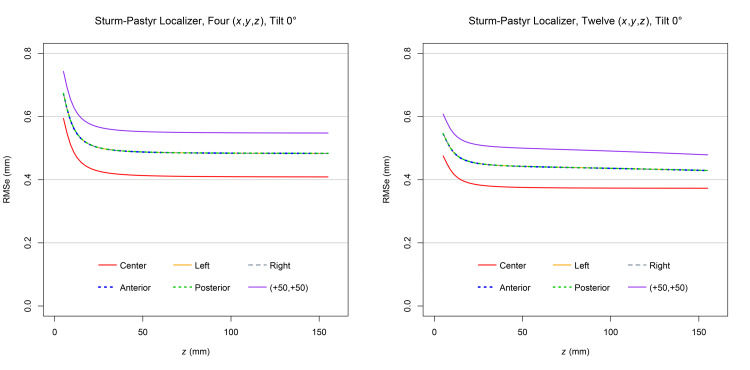
Plots of RMSe vs height $\left( z \right)$ at a $0^{\large \circ}$ tilt for four and 12 sets of Sturm-Pastyr $\left( x,y,z \right)$ coordinates The RMSe for four sets of $\left( x,y,z \right)$ coordinates exceeds the RMSe for 12 sets of $\left( x,y,z \right)$ coordinates at each target point for the range of heights $5.0 \mathrm{mm} \le z \le 155.0 \mathrm{mm}$. The plots for the anterior, posterior, left, and right target points are superimposed. RMSe: Root mean square error

Figure 5 demonstrates that for each target point and at a $0^{\large\circ}$ tilt, the RMSe predicted for four sets of $\left( x,y,z \right)$ coordinates exceeds the RMSe predicted for 12 sets of $\left( x,y,z \right)$ coordinates. Comparison to Figure 3 reveals that a fourth SP localizer decreases the RMSe for each target point; in particular, to a greater extent for the posterior target point than for the other target points. (Note that the range of the RMSe axis is $\left[0.0 \mathrm{mm}, 1.2 \mathrm{mm} \right]$ for Figure 3 but $\left[0.0 \mathrm{mm}, 0.8 \mathrm{mm} \right]$ for Figure 5.) The RMSe for the anterior, posterior, left, and right target points are all equal, as expected for a $0^{\large\circ}$ tilt and four SP localizers positioned symmetrically (i.e., at $90^{\large\circ}$ intervals) around the circumference of the ZD stereotactic frame. For each target point, the RMSe increases significantly as the height $\left( z \right)$ approaches 0.0mm inferiorly near the apices of the V-shaped SP localizers [3].

**Figure 6:**
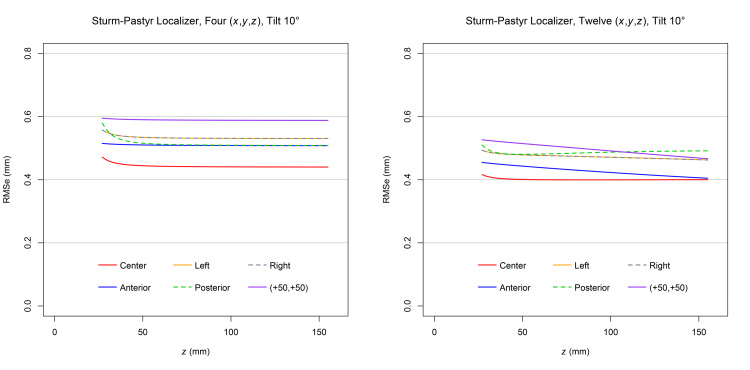
Plots of RMSe vs height $\left( z \right)$ at a $10^{\large \circ}$ tilt for four and 12 Sets of Sturm-Pastyr $\left( x,y,z \right)$ coordinates The RMSe for four sets of $\left( x,y,z \right)$ coordinates exceeds the RMSe for 12 sets of $\left( x,y,z \right)$ coordinates at each target point for the range of heights $27.0 \mathrm{mm} \le z \le 155.0 \mathrm{mm}$. The plots for the left and right target points are superimposed. RMSe: Root mean square error

Figure 6 demonstrates that for each target point and at a $10^{\large\circ}$ tilt, the RMSe predicted for four sets of $\left( x,y,z \right)$ coordinates exceeds the RMSe predicted for 12 sets of $\left( x,y,z \right)$ coordinates. Comparison to Figure 4 reveals that a fourth SP localizer decreases the RMSe for each target point; in particular, to a greater extent for the posterior target point than for the other target points. (Note that the range of the RMSe axis is $\left[0.0 \mathrm{mm}, 1.2 \mathrm{mm} \right]$ for Figure 4 but $\left[0.0 \mathrm{mm}, 0.8 \mathrm{mm} \right]$ for Figure 6.) The RMSe for the posterior target point exceeds the RMSe for the anterior target point, as expected for a $10^{\large\circ}$ anteroposterior tilt for which the CT scan plane intersects the anterior SP localizer at a greater height $\left( z \right)$ than it intersects the posterior SP localizer. (The accuracy of the V-shaped SP localizer is greater superiorly than inferiorly [3].) Comparison to Figure 5 reveals that for each target point, the RMSe for a $10^{\large\circ}$ tilt exceeds the RMSe for a $0^{\large\circ}$ tilt. The minimum allowed height $\left( z \right)$ of the CT scan plane is greater for a $10^{\large\circ}$ tilt than for a $0^{\large\circ}$ tilt so that the CT scan plane does not intersect the base of the ZD stereotactic frame posteriorly.

In view of the improved accuracy predicted for the attachment of a fourth SP localizer to the posterior aspect of the ZD stereotactic frame, the accuracy of the four SP localizer configuration was compared to the accuracy of a four N-localizer configuration that is the most accurate of all N-localizer configurations [5]. In this four N-localizer configuration, an N-localizer is attached to each of the anterior, posterior, left lateral, and right lateral aspects of a stereotactic frame in a configuration similar to the attachment of four SP localizers to the ZD frame.

For the four SP localizers, the tilt angle was $10^{\large \circ}$. For the four N-localizers, the tilt angle was $0^{\large \circ}$. The accuracy of the N-localizer is insensitive to tilt angle [3]; hence, the comparison of four N-localizers to four SP localizers does not require that the tilt angle for the N-localizers equal the tilt angle for the SP localizers.

The MC simulation for the four SP localizers employed 12 sets of $\left( x,y,z \right)$ coordinates. The MC simulation for the four N-localizers employed four sets of $\left( x,y,z \right)$ coordinates and eight sets of $\left( x,y \right)$ coordinates. The N-localizer is so-named because it comprises one diagonal rod and two vertical rods that form an N-shape [4]. Each diagonal rod provides one set of $\left( x,y,z \right)$ coordinates. Each vertical rod provides one set of $\left( x,y \right)$ coordinates that improve the accuracy in $x$ and $y$ but not in $z$, unlike a set of $\left( x,y,z \right)$ coordinates that improve the accuracy in $x$, $y$, and $z$ [5].

**Figure 7:**
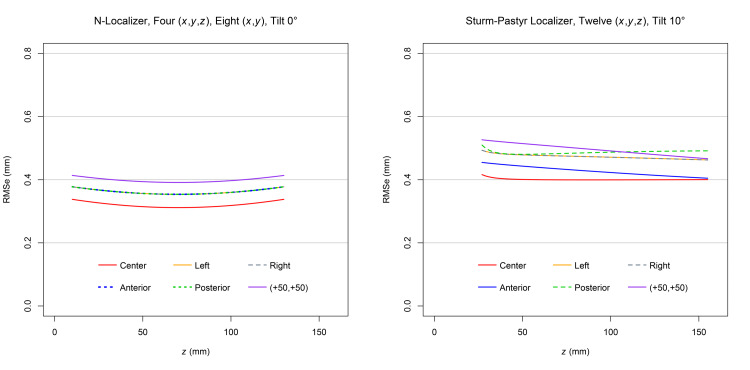
Plots of RMSe vs height $\left( z \right)$ for four N-localizers at a $0^{\large \circ}$ tilt and four Sturm-Pastyr localizers at a $10^{\large \circ}$ tilt For four N-localizers, four sets of $\left( x,y,z \right)$ coordinates and eight sets of $\left( x,y \right)$ coordinates are used to calculate the RMSe. For four Sturm-Pastyr (SP) localizers, 12 sets of $\left( x,y,z \right)$ coordinates are used to calculate the RMSe. The RMSe of the SP localizer for the range of heights $27.0 \mathrm{mm} \le z \le 155.0 \mathrm{mm}$ exceeds by 0.1mm the RMSe of the N-localizer for the range of heights $10.0 \mathrm{mm} \le z \le 130.0 \mathrm{mm}$. For the N-localizer, the plots for the anterior, posterior, left, and right target points are superimposed. For the SP localizer, the plots for the left and right target points are superimposed. RMSe: Root mean square error

Figure 7 demonstrates that for each target point, the RMSe of four SP localizers exceeds the RMSe of four N-localizers by only 0.1mm. This result is noteworthy, given that the SP localizer is significantly more susceptible to error than the N-localizer [3]. The greater susceptibility of the SP localizer is a consequence of its V-shape that diminishes the distances between fiducials in a CT scan image produced by a CT scan slice that is tilted or that intersects the SP localizer inferiorly near its apex [3]. The error caused by this physical limitation of the SP localizer may be mitigated but not eliminated, as demonstrated by Figures 3, 4, 5, 6.

## Conclusions

Monte Carlo (MC) simulation for the Zamorano-Dujovny (ZD) stereotactic frame predicts that the accuracy of the Sturm-Pastyr (SP) localizer may be improved by (1) calculating three sets of $\left( x,y,z \right)$ coordinates instead of only one set of $\left( x,y,z \right)$ coordinates for each SP localizer, (2) attaching four instead of three SP localizers to the ZD frame, (3) avoiding CT scanning inferiorly near the apices of the V-shaped SP localizers, and (4) limiting the tilt of the base of the ZD frame to 10$^{\large \circ}$ or less relative to the CT scan slice. When these four techniques are applied in concert, the improved accuracy of the SP localizer approaches the accuracy of the N-localizer.

## Figures and Tables

**Figure 8:**
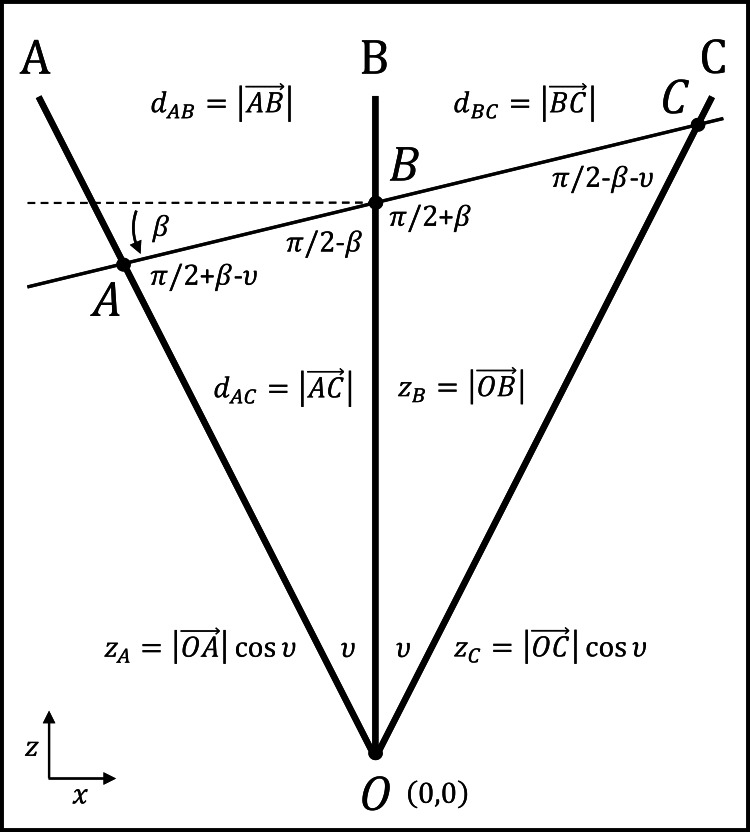
Depiction of the Sturm-Pastyr localizer The Sturm-Pastyr localizer is depicted by rods $\mathrm A$, $\mathrm B$, and $\mathrm C$ whose cylindrical axes intersect the CT scan plane at points $A$, $B$, and $C$ respectively [3]. The $z$-component of the Euclidean distance between points $O$ and $A$ is $z_A$. The $z$-component of the Euclidean distance between points $O$ and $C$ is $z_C$. The angle $\beta$ represents the tilt of the CT scan plane relative to the base of the stereotactic frame. The angle $\upsilon = \tan^{-1}\left ( 1/2 \right )$ is constant [1-3].

## Appendices

Figure 8 depicts a Sturm-Pastyr (SP) localizer that lies in an $\left( x,z \right)$ plane of constant $y$. The appendices of [3] extend the SP mathematical formulation presented by Dai, et al. [2] and thereby obtain equations (A9) and (A12) of those appendices that are used to derive equations (10-12) of these appendices.

The $z$-coordinate $z_B$ of the point $B$ where the CT scan plane intersects the cylindrical axis of rod $\mathrm B$ is given by equation (A12) from [3].

$$z_B = \frac{4d_{AB}d_{BC}}{\sqrt{\left ( d_{BC} + d_{AB} \right )^2 + 4 \left ( d_{BC} - d_{AB} \right )^2}} \;\;\;\; \;\; \left(2 \right)$$

The $x$-coordinate $x_B$ of the point $B$ equals zero.

The $\left( x_A, z_A \right)$ coordinates of the point $A$ where the CT scan plane intersects the cylindrical axis of rod $\mathrm A$ are obtained via the law of sines 

$$\frac{d_{AB}}{\sin \upsilon} = \frac{d_{OA}}{\sin \left ( \frac{\pi}{2} - \beta \right )} = \frac{d_{OA}}{\sin \left( \frac{\pi}{2}\right ) \cos \left( -\beta \right ) + \cos \left( \frac{\pi}{2}\right ) \sin \left( -\beta \right )} = \frac{d_{OA}}{\cos \beta} \;\;\;\;\;\; \left ( 3 \right )$$

where $\cos \beta$ is given by equation (A9) from [3].

$$\cos \beta = \frac{d_{BC} + d_{AB} }{\sqrt{\left ( d_{BC} + d_{AB} \right )^2 + 4 \left ( d_{BC} - d_{AB} \right )^2}} \;\;\;\;\;\; \left ( 4 \right )$$

Substituting equation (4) into equation (3) and rearranging yields $d_{OA}$.

$$d_{OA} = \frac{d_{AB}}{\sin \upsilon}\frac{d_{BC} + d_{AB}}{\sqrt{\left ( d_{BC} + d_{AB} \right )^2 + 4 \left ( d_{BC} - d_{AB} \right )^2}} \;\;\;\;\;\; \left ( 5 \right )$$

Substituting equation (5) into the equations $x_A = -d_{OA} \sin \upsilon$ and $z_A = d_{OA} \cos \upsilon$ and then substituting $\tan \upsilon = 1/2$ into the resulting equation for $z_A$ yields the $\left( x_A, z_A \right)$ coordinates.

$$x_A = -\frac{d_{AB} \left( d_{BC} + d_{AB} \right )}{\sqrt{\left ( d_{BC} + d_{AB} \right )^2 + 4 \left ( d_{BC} - d_{AB} \right )^2}} \;\;\;\;\;\; z_A = \frac{2 d_{AB} \left( d_{BC} + d_{AB} \right )}{\sqrt{\left ( d_{BC} + d_{AB} \right )^2 + 4 \left ( d_{BC} - d_{AB} \right )^2}} \;\;\;\;\;\; \left ( 6 \right )$$

 The $\left( x_C, z_C \right)$ coordinates of the point $C$ where the CT scan plane intersects the cylindrical axis of rod $\mathrm C$ are obtained via the law of sines 

$$\frac{d_{BC}}{\sin \upsilon} = \frac{d_{OC}}{\sin \left ( \frac{\pi}{2} + \beta \right )} = \frac{d_{OC}}{\sin \left( \frac{\pi}{2}\right ) \cos \beta  + \cos \left( \frac{\pi}{2}\right ) \sin \beta } = \frac{d_{OC}}{\cos \beta} \;\;\;\;\;\; \left ( 7 \right )$$

where $\cos \beta$ is given by equation (4).

Substituting equation (4) into equation (7) and rearranging yields $d_{OC}$.

$$d_{OC} = \frac{d_{BC}}{\sin \upsilon}\frac{d_{BC} + d_{AB}}{\sqrt{\left ( d_{BC} + d_{AB} \right )^2 + 4 \left ( d_{BC} - d_{AB} \right )^2}} \;\;\;\;\;\; \left ( 8 \right )$$

Substituting equation (8) into the equations $x_C = d_{OC} \sin \upsilon$ and $z_C = d_{OC} \cos \upsilon$ and then substituting $\tan \upsilon = 1/2$ into the resulting equation for $z_C$ yields the $\left( x_C, z_C \right)$ coordinates.

$$x_C = \frac{d_{BC} \left( d_{BC} + d_{AB} \right )}{\sqrt{\left ( d_{BC} + d_{AB} \right )^2 + 4 \left ( d_{BC} - d_{AB} \right )^2}} \;\;\;\;\;\; z_C = \frac{2 d_{BC} \left( d_{BC} + d_{AB} \right )}{\sqrt{\left ( d_{BC} + d_{AB} \right )^2 + 4 \left ( d_{BC} - d_{AB} \right )^2}} \;\;\;\;\;\; \left ( 9 \right )$$

Inspection of equations (2), (6), and (9) reveals that the three sets of $\left( x, z \right)$ coordinates $\left( x_A, z_A \right)$, $\left( 0, z_B \right)$, and $\left( x_C, z_C \right)$ are expressed in terms of only the two Euclidean distances $d_{AB}$ and $d_{BC}$; hence, these three sets of $\left( x, z \right)$ coordinates are linearly dependent. However, these sets of $\left( x, z \right)$ coordinates become linearly independent when calculated via the following equations obtained by substituting $d_{AC} = d_{AB} + d_{BC}$ into equations (2), (6), and (9).

$$z_B = \frac{4d_{AB}d_{BC}}{\sqrt{d_{AC}^2 + 4 \left ( d_{BC} - d_{AB} \right )^2}} \;\;\;\; \;\; \left( 10 \right) \\ x_A = -\frac{d_{AB} d_{AC}}{\sqrt{d_{AC}^2 + 4 \left ( d_{BC} - d_{AB} \right )^2}} \;\;\;\;\;\; z_A = \frac{2 d_{AB} d_{AC}}{\sqrt{d_{AC}^2 + 4 \left ( d_{BC} - d_{AB} \right )^2}} \;\;\;\;\;\; \left ( 11 \right ) \\ x_C = \frac{d_{BC} d_{AC}}{\sqrt{d_{AC}^2 + 4 \left ( d_{BC} - d_{AB} \right )^2}} \;\;\;\;\;\; z_C = \frac{2 d_{BC} d_{AC}}{\sqrt{d_{AC}^2 + 4 \left ( d_{BC} - d_{AB} \right )^2}} \;\;\;\;\;\; \left ( 12 \right )$$

The three Euclidean distances $d_{AB}$, $d_{BC}$, and $d_{AC}$ become linearly independent when image noise randomly perturbs the $\left( u,v \right)$ coordinates of the centers of the fiducials $A$, $B$, and $C$ depicted in Figure 1, as explained in the technical report. Hence, equations (10-12) are linearly independent because they express the three sets of $\left( x, z \right)$ coordinates $\left( x_A, z_A \right)$, $\left( 0, z_B \right)$, and $\left( x_C, z_C \right)$ in terms of the three linearly independent Euclidean distances $d_{AB}$, $d_{BC}$, and $d_{AC}$. The numerators of equations (10-12) contain the products $d_{AB} d_{BC}$, $d_{AB} d_{AC}$, and $d_{BC} d_{AC}$ for the points of intersection $B$, $A$, and $C$ respectively.
